# Phospholipase A2 regulation of bovine endometrial (BEND) cell prostaglandin production

**DOI:** 10.1186/1477-7827-6-44

**Published:** 2008-09-23

**Authors:** James D Godkin, Mary P Roberts, Mona Elgayyar, Wei Guan, Patricia K Tithof

**Affiliations:** 1 Department of Animal Science, The University of Tennessee, Knoxville, TN, USA; 2 The University of Tennessee College of Veterinary Medicine, Department of Pathology, Knoxville, TN, USA

## Abstract

**Background:**

Prostaglandins (PG), produced by the uterine endometrium, are key regulators of several reproductive events, including estrous cyclicity, implantation, pregnancy maintenance and parturition. Phospholipase A2 (PLA2) catalyzes the release of arachidonic acid from membrane phospholipids, the rate-limiting step in PG biosynthesis. The bovine endometrial (BEND) cell line has served as a model system for investigating regulation of signaling mechanisms involved in uterine PG production but information concerning the specific PLA2 enzymes involved and their role in regulation of this process is limited. The objectives of this investigation were to evaluate the expression and activities of calcium-dependent group IVA (PLA2G4A) and calcium-independent group VI (PLA2G6) enzymes in the regulation of BEND cell PG production.

**Methods:**

Cells were grown to near-confluence and treated with phorbol 12, 13 dibutyrate (PDBu), interferon-tau (IFNT), the PLA2G4A inhibitor pyrrolidine-1 (PYR-1), the PLA2G6 inhibitor bromoenol lactone (BEL) and combinations of each. Concentrations of PGF2alpha and PGE2 released into the medium were determined. Western blot analysis was performed on cellular protein to determine effects of treatment on expression of PLA2G4A, PLA2G6 and PLA2G4C. PLA2 assays were performed on intact cells by measuring arachidonic acid and linoleic acid release and group-specific PLA2 activity assays were performed on cell lysates.

**Results:**

BEND cells produced about 10-fold more PGE2 than PGF2alpha under resting conditions. Production of both PGs increased significantly in response to PDBu-stimulation. PYR-1 significantly diminished production of both PGs by resting cells and abolished the stimulatory effect of PDBu. BEL stimulated production of both PGs. IFNT reduced both PGE2 and PGF2alpha production by resting cells and diminished PDBu stimulation of PG production. Conversely, IFNT did not significantly reduce BEL stimulation of PG production. Cellular expression of PLA2G4A was enhanced by PDBu and this response was diminished by IFNT. Expression of PLA2G6 was not observed to be affected by treatments and no PLA2G4C expression was observed. Arachidonic acid release from intact cells was significantly increased by PDBu and this effect was attenuated by PYR-1 but not by BEL. Release of linoleic acid from intact cells was stimulated by PDBu and inhibited by BEL but not PYR-1. Group specific PLA2-activity assays demonstrated both PLA2G4A and PLA2G6 activity.

**Conclusion:**

Results from this study demonstrate that PGE2 and PGF2-alpha production by BEND cells is mediated by the activity and expression of PLA2G4A. Interferon-tau treatment diminished expression of PLA2G4A and PG production. BEND cells were shown to express PLA2G6 but, unlike primary or early passage luminal bovine endometrial cells, stimulation of PLA2G6 activity was not associated with increased PG production.

## Background

Prostaglandins, produced by the endometrial epithelium, are important regulators of several reproductive processes, including estrous cyclicity, implantation, pregnancy maintenance and parturition [1]. Prostaglandin (PG) biosynthesis is dependent on arachidonic acid (AA) release from membrane phospholipids catalyzed by phospholipase A_2 _enzymes [reviewed in [2]]. Arachidonic acid is then metabolized to intermediate products, PGG_2 _and PGH_2_, by a cyclooxygenase reaction and by a peroxidase reaction, respectively, both performed by cyclooxygenase (COX) -1 and/or -2. Prostaglandin H_2 _is converted to bioactive prostaglandins, such as PGF_2α_, PGE_2_, PGD_2 _and PGI_2_, by terminal PG synthases, which may exhibit tissue specific distribution [3].

Bovine and ovine endometrial explants and epithelial cell cultures have proven to be functional models for analysis of pathways that regulate PG biosynthesis. Early studies used endometrial explants [4,5] or glandular endometrial epithelial cells [5-7] harvested from animals at late diestrus. More recent studies have utilized primary or early passage luminal epithelial (LE) cells collected from animals early in the cycle (days 1–4) because the luminal epithelium is the major site of endometrial PG production and these cells exhibit much better growth characteristics than LE cells collected during diestrus [8-11]. Results from experiments with explants and glandular or luminal epithelial cells are consistent; oxytocin stimulates PGF_2α _and PGE_2 _production and interferon-tau (IFNT) diminishes this response. Bovine endometrial epithelial cells produce greater quantities of PGF_2α _than PGE_2 _and the PGF_2α _response to oxytocin stimulation is stronger. The cellular response to IFNT, alone, is biphasic. Low concentrations (< 1 μg/ml) of IFNT diminish basal PG production and high concentrations (>1 μg/ml) stimulate PG production [10]. Interestingly, both low and high concentrations of IFNT diminish oxytocin stimulated PG production. Agonist-stimulated PG production, by oxytocin or high concentrations of IFNT, is associated with increased expression of COX-2 mRNA and protein [9,10], prostaglandin E_2 _synthase (PGES) [10], and prostaglandin F_2α _synthase (PGFS) mRNA [11]. Attenuation of oxytocin-stimulated PGF_2α _production is associated with decreased COX-2 production [9,10] and alteration in the expression of terminal PGF synthase [11].

Numerous studies on the regulation of PG biosynthesis have been performed with the BEND, the bovine endometrial cell line [reviewed in [12]]. BEND cells have been described as spontaneously immortalized endometrial cells originally isolated from a cow on day 14 of the cycle [13]. They have been described to have both epithelial-like [12,13] and stromal-like [14] morphological characteristics. BEND cells produce significantly more (10–20 fold) PGE_2 _than PGF_2α _under both resting and stimulated conditions, and they respond to phorbol ester (phorbol 12-myristate 13-acetate, PMA, or phorbol 12,13 dibutyrate, PDBu) stimulation, but not oxytocin stimulation, with increased PG production [12,14,15]. Similar to primary endometrial epithelial cells, BEND cells increase expression of COX-2 mRNA [16], protein and PGFS [15] when PG production is stimulated and these responses are diminished by IFNT at concentration less than 5 μg/ml [15]. Unlike primary endometrial epithelial cells, BEND cells are not stimulated to produce PGs by high levels of IFNT alone and when treated with both PDBu and high levels of IFNT (>5–20 μg/ml) inhibitory activities of IFNT on PG production are ameliorated [15].

Despite the fact that phospholipase A_2 _(PLA_2_) catalyzes the release of arachidonic acid, the rate-limiting step in PG biosynthesis, most studies on the regulation of uterine PG production have focused on expression and activation of downstream enzymes such as cyclooxygenases, which catalyze the committed step, and PG synthases. The reports [6,9,16] that have included analysis of PLA_2 _expression in domestic ruminant uterine PG production, focused on a single enzyme, cPLA_2α_, (Group IVA PLA_2 _or PLA2G4A). Interestingly, while none of these studies identified consistent significant changes in expression of protein or mRNA for PLA2G4A in association with alterations in PG production, non-specific PLA_2 _inhibitors significantly diminished basal and agonist-induced PG production [6,9,17].

Mammalian PLA_2_s constitute a growing family of enzymes, currently containing about 20 members, that catalyze hydrolysis of fatty acids from the *sn*-2 position of phospholipids with the concomitant formation of lysophospholipid [18]. Based largely on structural and enzymatic properties, PLA_2_s have been grouped into four main subfamilies: low molecular weight secretory PLA_2_s (PLA2 Groups 1–3, 5, 10 and 12); cytosolic PLA2G4; calcium independent PLA2G6; and platelet activating factor (PAF) acid hydrolases (PLA2G7 and PLA2G8). The intracellular PLA2G4 and PLA2G6 enzymes are of particular interest in the regulation of uterine PG biosynthesis because their sites of action are the perinuclear membranes where downstream AA metabolizing enzymes reside.

Recently, we characterized PLA2G4A, PLA2G4C and PLA2G6 activity and expression in early passage bovine luminal endometrial epithelial cells [19]. It was observed that oxytocin stimulation of PGF_2α _production was associated with increased expression and activity of PLA2G6 and IFNT diminished these responses. In addition, the PLA2G6 inhibitor, bromoenol lactone (BEL), abolished oxytocin stimulation of PGF_2α _production. Results indicated that oxytocin stimulation of endometrial PGF_2α _production is mediated, at least in part, through activation of PLA2G6.

Both BEND cells and primary cultures of endometrial epithelial cells have served as models for regulation of uterine prostaglandin biosynthesis and the mechanism by which IFNT modifies this process. Physiological differences between the two cell types have been identified [14]. The objective of the present study was to determine the role of PLA_2 _enzymes in BEND cell PG production and relate these results to the reported differences between BEND cells and primary endometrial epithelial cells.

## Methods

BEND cells were a generous gift from Dr. T. Hansen (Colorado State University, Ft Collins) and are commercially available from American Type Culture Collection (ATCC no. CRL-2398, Manassas, VA). Dulbecco minimum essential medium (MEM), Ham's F-12, Hanks' balanced salt solution (HBSS), gentamicin, oxytocin, PDBu, bovine serum albumin, fetal bovine serum, horse serum, aprotinin, leupeptin and pepstatin were from Sigma Chemical Co (St Louis, MO). Enzyme Immunoassay kits for PGE_2 _(catalog no. 900-001) and PGF_2α _(catalog no. 901-069) were from Assay Designs, Inc (Ann Arbor, MI). 1-palmitoyl-2-(1-^14^C) arachidonyl-phosphotidylcholine (^14 ^C-AA-PC), 1-palmitoyl-2-(1-^14^C) linoleoyl-phosphotidylcholine (^14^C-LA-PC), ^3^H-arachidonic acid and ^3^H-linoleic acid were from American Radiolabeled Chemicals, (St. Louis, MO). Group IVA PLA_2 _antibody (SC-454) was from Santa Cruz Biotechnology, Inc (Santa Cruz, CA), Group VI PLA_2 _antibody (Anti-iPLA_2_, #07-169) was from Upstate Cell Signaling Solutions (Lake Placid, NY) and the anti-Group IVC was a gift from Dr. C.C. Leslie (University of Colorado, Boulder, CO). Recombinant ovine IFNT (antiviral activity, 1 × 10 ^8 ^U/mg) was donated kindly by Dr. F.W. Bazer (Texas A&M University, College Station, TX). Pyrrolidine-1 (PYR-1) was a generous gift of Dr. Michael Gelb (University of Washington, Seattle WA). Tissue culture plastic wares were from Becton Dickinson and Co. (Franklin Lakes, NJ). Unless noted otherwise, additional chemicals were from Sigma Chemical Corp.

### Cell culture

BEND cells were cultured and propagated by the method described by Staggs, et al. [13], with modest modifications. Briefly, cells were seeded into tissue culture dishes of the appropriate size for each experiment (see below) at a concentration of 0.5 × 10^5 ^cells per ml in culture medium (40% Ham's F-12, 40% MEM, 200 U insulin/L, 50 μg gentamicin, 10% FBS, 10% horse serum) at 37°C in a humidified atmosphere of 95% air and 5% CO_2 _and culture medium was changed every other day. For all experiments, cells were grown to 80–90% confluence before application of treatments which were applied in triplicate and each experiment was repeated (n = 6) unless noted otherwise.

### PG assays

Cells were grown in 6-well plates, washed three times with HBSS and cultured for 6 h in unsupplemented Ham's F-12/MEM with the following treatments: vehicle control, PDBu (100 ng/ml), IFNT (50 ng/ml), the PLA2G6 inhibitor BEL (7.5 μM), the group PLA2G4A inhibitor PYR-1 (0.4 μM), PDBu + IFNT, PDBu + BEL, IFNT + BEL, PDBu + PYR-1. Vehicles, Ham's F-12/MEM for IFNT, and DMSO for PDBu and the inhibitors, were added to each well. At the end of the incubation period, medium was harvested and stored frozen at -200 C. Prostaglandin F2α and E2 assays were performed with enzyme immunoassay kits from Assay Designs, Inc., according to supplier's instructions. Inter- and intraassay coefficients of variation (n = 12) were 9% and 11%, respectively.

### Western blot analysis

Western blot analysis was performed as described previously [20]. Briefly, cells were grown in 60 mm dishes and treated with vehicle (control), PDBu and IFNT (50 ng and 1000 ng), alone and in combination for 6 h as described above. Cells were lysed in RIPA buffer (50 mM Tris-HCl, 150 mM NaCl, 1% SDS, 0.5% sodium deoxycholate, 1 mM DTT, 100 μM PMSF, 1% NP-40), and 1 × protease inhibitor cocktail (Roche Applied Science, Indianapolis, IN). Lysates from triplicate treatment wells were combined, sonicated and centrifuged (10,000 g, 20 minutes) and supernatants were subjected to 10% SDS-polyacrylamide gel electrophoresis and transferred onto nitrocellulose membranes. Non-specific binding sites were blocked with 5% non-fat dry milk and membranes were incubated with antibodies to PLA2G4A, PLA2G4C, and PLA2G6 in 1:800, 1:400 and 1:1000 dilutions, respectively, overnight at 4°C. Membranes were rinsed 3 times and protein bands were visualized by enhanced chemiluminescence (Amersham Biosciences, Arlington Heights, IL). A blot was prepared from each of the replicated experiments.

### ^3^H-arachidonic acid and ^3^H-linoleic acid released from intact cells

Intact cell PLA_2 _assays were performed on cells prelabeled for 24 h with either ^3^H-arachidonic acid (^3^H-AA; 0.25 μCi/ml) or ^3^H-linoleic acid (^3^H-LA, 0.25 μCi/ml). Cells were washed three times with Hank Balanced Salt Solution (HBSS), equilibrated for 45 min and treatments applied for 1 hr. Cells were treated with 100 ng/ml PDBu in the presence and absence of 7.5 μM BEL or 0.4 μM PYR-1. Inhibitors were added 10 minutes prior to treatment with PDBU. Medium and lysed cells were harvested separately and % of total radioactivity released into the medium determined by scintillation counting.

### PLA_2 _activity in cellular homogenates

PLA_2 _assays were performed as described previously [20]. Briefly, cells were washed and treated with either the PLA2G4A inhibitor, PYR-1 (0.4 μM) or the PLA2G6 inhibitor BEL (7.5 μM) for 3 hours. At the end of the incubation period, cells were washed with Ca^++^-free PBS containing 5 mM EGTA and 1 mM PMSF, lysed in homogenizing buffer (50 mM Tris HCl, pH 7.4, 0.5 mM dithiothreitol, 20% glycerol, 1 μg/ml leupeptin, 10 μg/ml aprotinin, 1 mM phenylmethylsulfonylchloride) and sonicated two times for 10 seconds on ice. The substrates 1-palmitoyl-2-[arachidonoyl] phosphatidylcholine (cold AA-PC) and 1-palmitoyl-2-[arachidonoyl-1-^14^C] phosphatidylcholine (^14^C-AA-PC) or 1-palmitoyl-2-[linoleoyl] phosphatidylcholine (cold LA-PC), 1-palmitoyl-2-[linoleoyl-1-^14^C] phosphatidlycholine (^14^C-LA-PC) were used to assay for PLA_2 _activity. The substrates were dried under nitrogen and resuspended by sonication in assay buffer (10 mM Hepes, pH 7.5) to a final optimum concentration of cold to radiolabeled substrate as determined previously. Calcium-dependent activity (primarily PLA2G4A) assays were performed with AA-PC and ^14^C-AA-PC in the presence of 5 mM CaCl_2 _and calcium-independent activity (primarily PLA2G6 and PLA2G4C) assays were performed using the same substrates but in the absence of CaCl_2 _and the presence of EGTA (5 mM). Assays optimized for identification of PLA2G6 utilized LA-PC and ^14^C-LA-PC in both the presence and absence of Ca^++ ^and presence of 400 μM Triton-X and 0.8 mM ATP which enhance the activity of this enzyme [29]. Experiments were terminated by addition of chloroform-methanol, 2:1 (v/v), the chloroform layer was extracted and lipids separated by thin-layer chromatography (hexane: diethyl ether: glacial acetic acid, 7:3:0.2). Lipids were visualized with I_2 _vapor, the zones corresponding to fatty acid and phospholipids were excised and radioactivity determined by scintillation counting.

### Statistical analysis

Data for PG production and PLA_2 _activity are presented as means +/- SEM and subjected to least squares analysis of variance using the general linear models procedure of the Statistical Analysis System (SAS Institute, Cary, NC). Sources of variance included experiments, treatments and their interactions. Individual comparisons of means were made using Student-Neuman-Keuls test in which the independent variables were the treatments and the dependent variables were the levels of PG or PLA_2 _activity produced. Differences were considered statistically significant when p < 0.05.

## Results

### Prostaglandin production

**Figure 1:**
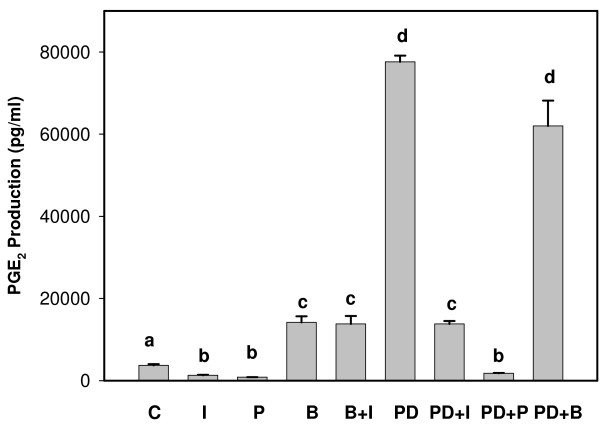
**Prostaglandin E_2 _production by BEND cells**. Cell culture medium was harvested 6 h after addition of treatments and concentrations of PGE_2 _were analyzed by ELISA. Treatments included: Control (C), IFNT (I), Pyrrolodine-1 (P), Bromoenol lactone (B), B +I, Phorbol 12, 13 dibutyrate (PD), PD + I, PD + P and PD + B. Treatments were performed in triplicate and repeated (n = 6). Data are expressed as means +/- SEM. Columns with different superscripts are significantly different (p < 0.05).

**Figure 2:**
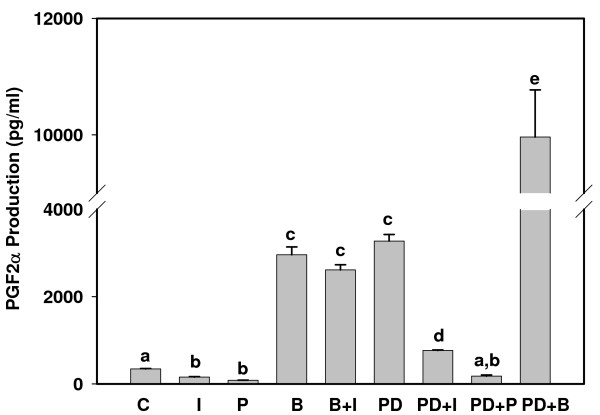
**Prostaglandin F_2α _production by BEND cells**. Cell culture medium was harvested 6 h after addition of treatments and concentrations of PGF_2α _were analyzed by ELISA. Treatments included: Control (C), IFNT (I), Pyrrolodine-1 (P), Bromoenol lactone (B), B + I, Phorbol 12, 13 dibutyrate (PD), PD + I, PD + P and PD + B. Treatments were performed in triplicate and repeated (n = 6). Data are expressed as means +/- SEM. Columns with different superscripts are significantly different (p < 0.05).

Production of PGE_2 _and PGF_2α _are illustrated in Figures 1 and 2, respectively. BEND cells produced about 10-fold more PGE_2 _than PGF_2α _under resting conditions. Production of both PGs increased significantly (10- to 20-fold) in response to PDBu stimulation, which had a greater effect on PGE_2 _production. PYR-1, the PLA2G4A inhibitor, significantly diminished production of both PGs by resting cells and abolished the stimulatory effect of PDBu. Interestingly, BEL, the PLA2G6 inhibitor, stimulated production of both PGs in control cells and PGF_2α _release in PDBu-treated cells. IFNT reduced both PGE_2 _and PGF_2α _production from resting cells and diminished PDBu stimulation of PG production. Conversely, IFNT did not significantly reduce BEL stimulation of PG production.

### PLA_2 _protein expression

**Figure 3:**
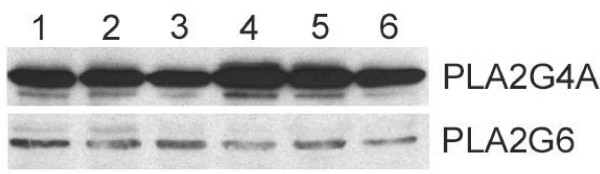
**BEND cell expression of PLA2G4A and PLA2G6**. BEND cells were treated for 6 h with vehicle (control, lane 1) 50 ng IFNT (lane 2), 1000 ng IFNT (lane 3), PDBu (lane 4), PDBu + 50 ng IFNT (lane 5) or 1000 ng IFNT + PDBu (lane 6). Western blot analyses were performed on cellular proteins using antibodies against A, PLA2G4A and B, PLA2G6 as described in Methods.

Western blot analysis of cellular proteins from BEND cells demonstrated that the PLA2G4A antibody recognized a protein that migrated at ~110 kD and a very minor protein of slightly less mass (~107 kD) that may have been a breakdown product (Figure 3, top). The PLA2G6 antibody cross-reacted with a protein that migrated at ~85 kD (Figure 3, bottom). The PLA2G4C antibody did not detect any major protein bands (not shown). Two isoforms of PLA2G6 were identified, a major band at 85 kD and a minor band of slightly greater mass (~88 kD). Similar results have been observed previously and are likely the result of alternative splicing of the PLA2G6 transcript resulting in multiple isoforms of the enzyme. Cells were treated for 6 h with vehicle (control, lane 1), IFNT (50 ng/ml, lane 2), IFNT (1000 ng/ml, lane 3), PDBu (lane 4), PDBu plus IFNT (50 ng/ml, lane 5) or PDBu plus IFNT (1000 ng/ml, lane 6) to determine effects of treatments on PLA_2 _isoform expression. As illustrated in the representative Figure 3, IFNT, alone, had little effect on expression of PLA2G4A when compared to control. In contrast PDBu stimulated PLA2G4A expression and IFNT, when combined with PDBu, diminished the stimulatory effect. Conversely, treatments had little effect on expression of PLA2G6.

### ^3^H-arachidonic acid release and PLA_2 _activity assays

**Figure 4:**
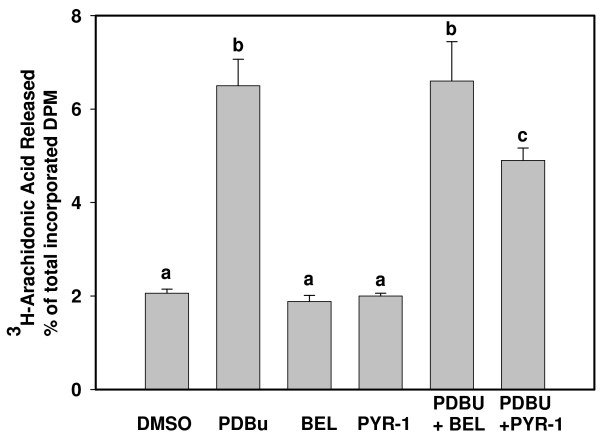
**Arachidonic acid release by BEND cells**. ^3^H-arachidonic acid (0.25 μCi/ml) was applied to cells for 24 h, cells were washed and treated for one h with vehicle (C, control), Phorbol 12,13 dibutyrate (PD), Bromoenol lactone (B), Pyrroline-1 (P), PD + B or PD + P. Medium and lysed cells were harvested separately and % of total radioactivity released into the medium determined by scintillation counting. Treatments were performed in triplicate and repeated (n = 6). Data are expressed as means +/- SEM. Columns with different superscripts are significantly different (p < 0,05).

**Figure 5:**
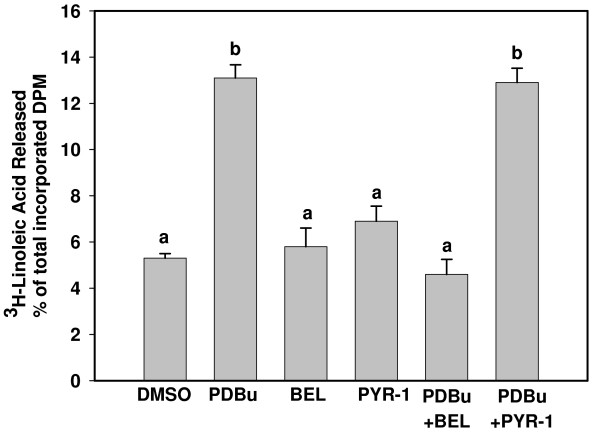
**Linoleic acid release by BEND cells**. ^3^H-linoleic acid (0.25 μCi/ml) was applied to cells for 24 h, cells were washed and treated for one h with vehicle, (C, control), Phorbol 12,13 dibutyrate (PD), Bromoenol lactone (B), Pyrrolodine-1 (P), PD + B or PD + P. Medium and lysed cells were harvested separately and % of total radioactivity released into medium determined by scintillation counting. Treatments were performed in triplicate and repeated (n = 6). Data are expressed as means +/- SEM. Columns with different superscripts are significantly different (p < 0.05).

Release of incorporated^3^H-arachidonic acid and ^3^H-linoleic acid were used as measures of PLA_2 _activity in intact cells because PLA2G4A is selective for arachidonic acid, whereas PLA2G6 is non-selective and will cleave both arachidonic acid and linoleic acid from membrane phospholipids. As can be seen in Figure 4, PDBu induced significant release of ^3^H-arachidonic acid when compared to DMSO control. This effect was attenuated by the Group IVA PLA_2 _inhibitor PYR-1, but not by the Group VI inhibitor, BEL. In contrast, PYR-1 had no effect on PDBu stimulation of ^3^H-linoleic acid release (Figure 5) but BEL inhibited this action.

**Figure 6:**
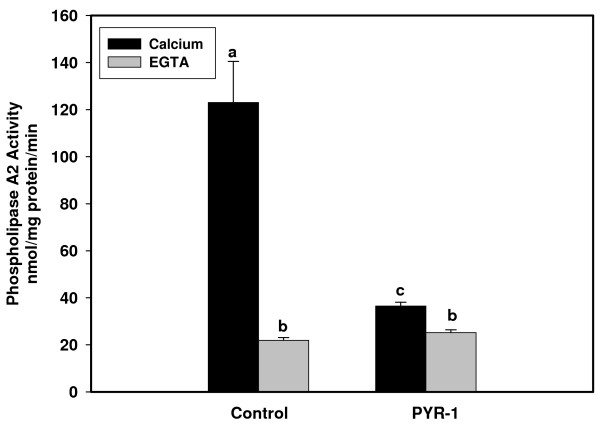
**PLA_2 _activity assays using AA-PC as substrate**. Ca^++^-dependent PLA_2 _activity assays were performed on cellular lysates with AA-PC and ^14^C-AA-PC in the presence of 5 mM CaCl_2 _and Ca^++^-independent assays were performed using the same substrate but in the absence of Ca^++ ^and the presence of 5 mM EGTA. Columns with different superscripts are significantly different (p < 0.05). The PLA2G4A inhibitor, pyrroline-1 (PYR-1) inhibited Ca^++^-dependent activity.

As can be seen in Figure 6, BEND cells exhibited significant PLA_2 _activity from cell lysates when ^14^C-PC-AA was used as a substrate both in the presence or absence of calcium; however, PLA_2 _activity was significantly greater in the presence of calcium than in all other groups. The PLA2G4A inhibitor, PYR-1, returned values to baseline in both the presence or absence of calcium.

**Figure 7:**
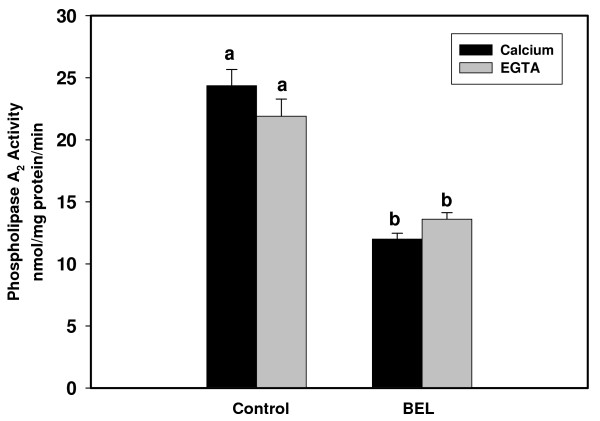
**PLA_2 _activity assays using LA-PC as substrate**. Ca^++^-dependent activity assays were performed on cellular lysates with LA-PC and ^14^C-LA-PC in the presence of 5 mM CaCl_2 _and Ca^++^-independent assays were performed using the same substrate but in the absence of Ca^++ ^and the presence of 5 mM EGTA. Columns with different superscripts are significantly different (p < 0.05). The PLA2G6-inhibitor, bromoenol lactone (BEL), was inhibitory to PLA_2 _activity toward LA-PC.

Cellular lysates also exhibited significant PLA_2 _activity when ^14^C-PC-LA was used as a substrate and this activity was not different in the presence or absence of calcium (Figure 7). The PLA2G6 inhibitor, BEL, attenuated activity in the presence of calcium and in the presence of EGTA.

## Discussion

The results from this investigation strongly indicate that PLA2G4A is the enzyme that liberates arachidonic acid for PG biosynthesis in BEND cells. In addition, the results suggest that stimulation of PG production is regulated at the level of PLA2G4A activity and expression. Several lines of evidence support the concept that PG production is regulated by PLA2G4A in BEND cells. One, the PLA2G4A inhibitor PYR-1, significantly diminished basal PG production and abolished PDBu stimulation of PG production and ^3^H-AA release from intact cells. Two, stimulation of PG production by PDBu was associated with increased expression and activity of PLA2G4A. Three, the predominant PLA_2 _activity in BEND cell lysates was calcium-dependent. Also, BEND cells demonstrated significantly more activity when ^14^C-PC-AA was used as a substrate when compared to ^14^C-PC-LA, suggesting that the predominant isoform active in these cells is arachidonoyl-selective, a characteristic of PLA2G4A. Finally, reduction of PG production by IFNT was associated with diminished PLA2G4A expression.

In addition to the demonstrated PLA2G4A activity, BEND cells exhibited considerable PLA2G6 activity. The use of ^14^C-PC-LA as a substrate, showed activity was not different in the presence or absence of calcium, consistent with calcium-independent activity. This activity was inhibited by BEL, suggesting activity of a PLA2G6 isoform. Similarly, BEL, but not PYR-1, inhibited incorporated ^3^H-linoleic acid release from intact cells. However, BEL was not inhibitory to BEND cell PG synthesis, an indication that PLA2G6 activation does not promote PG production in these cells. PLA2G6 has been shown to be involved in maintaining membrane homeostasis in some cell types [18] and it is feasible that it plays a similar role in BEND cells. Interestingly, inhibition of PLA2G6 activity with BEL resulted in increased production of both PGF2α and PGE2 and BEL was synergistic with PDBu in promoting production of PGF2α. In previous studies with primary or early passage luminal endometrial cells, we observed that BEL stimulated PGE2 production and inhibited PGF2α production [19]. Whereas BEND cell PGF2α and PGE2 production appears to be mediated by PLA2G4A, oxytocin-stimulated luminal endometrial cell PGF2α production was associated with activation of PLA2G6. In BEND cells, inhibition of PLA2G6 activity by BEL may result in greater concentrations of phospholipid substrate available for hydrolysis by PLA2G4A resulting in increased production of both PGF2α and PGE2.

Several studies have used BEND cells as a model system for investigation of signaling mechanisms involved in uterine cell PG production and IFNT inhibition of this process [reviewed in [12]]. Base on results from these studies, it was concluded that phorbol ester-stimulation of PG production and reduction of this effect, by IFNT, was mediated by positive and negative regulation of COX-2 expression. For example, Binelli et al. [16] reported that activation of the protein kinase C (PKC) pathway with PDBu increased COX-2 expression and PGF_2α _production. Pru et al. [23] observed that PDBu treatment stimulated COX-2 mRNA expression within 30–60 minutes, while COX-2 protein and PG production increased at 3 hr. Co-treatment of cells with IFNT and PDBu results in diminished COX-2 mRNA, COX-2 protein and PG production. One study [16] observed that PDBu tended to increase (p < 1.0) PLA_2 _protein expression and IFNT tended to diminish this response, but only after 12 hours of culture, but PLA_2 _activity was not evaluated. In the present study, we observed an increase in PLA_2 _at 6 hours, the only time point tested, and maximal rate of increase in PLA_2_activity at 3 hours. In preliminary studies, an hourly time course of PLA_2 _activity was performed (not shown) and significant increases were observed in the first hour reaching a maximum rate of increase at 3 hours. These results indicate that increases in PLA_2 _activity in response to PDBu is an early response, while the increase in PLA_2 _expression is a delayed response. Based on these, and previous results [19], it is suggested that uterine PG production is regulated, at least in part, at the level of PLA_2 _activity and expression.

Stimulation of BEND cells with PDBu results in the phosphorylation and activation of several components of mitogen activated protein kinase (MAPK) pathways [23,24]. It is well established in a variety of cell types that MAPKs phosphorylate and activate PLAG4A resulting in arachidonic acid release [2]. Arachidonic acid, in addition to being the major prostaglandin precursor, can act as a signaling molecule and affects intracellular Ca^++ ^concentrations [25]. Importantly, arachidonic acid up-regulates COX-2 expression and PG production in a variety of cell types [26,27]. Parent, et al. [28], demonstrated that exogenous arachidonic acid stimulates COX-2 expression followed by a 30-fold increase in PG production by bovine endometrial epithelial cells. In BEND cells, arachidonic acid enhanced PGF_2α _production in PDBu-stimulated cells [29]. It is suggested that PDBu-activation of the PKC/MAPK pathway results in increased activity and expression of PLA2G4A which liberates arachidonic acid from perinuclear membranes. Endogenous arachidonic acid then may serve both as substrate for, and inducer of, COX-2 which completes the committed step in PG biosynthesis. Our results demonstrating phorbol ester stimulation of PLA2G4A activity and expression do not preclude the possibility of similar actions on COX-2 expression. Hughes-Fulford et al. [27] suggested that activation of PLA2G4A expression may involve signaling mechanisms similar to those of COX-2, since the genes have several identical promoter elements and the genes are on the same region of the same chromosome, in mice and humans. The genes for PLA2G4 and COX-2 are located in the same region of chromosome 16 in cattle [30] and review of the promoter region revealed several promoter elements in common. This raises the possibility that the two genes may be co-regulated, a phenomenon observed by others [31].

The mechanism by which IFNT exerts negative regulation on PG production in BEND cells is unclear. Guzeloglu, et al, [24] investigated effects of IFNT on COX-2 mRNA stability and concluded that IFNT may diminish PDBu-stimulated PG production by accelerating COX-2 degradation, mediated through an unidentified transcription dependent mechanism. Thatcher, et al, [12] suggested that IFNT, acting through Type I IFN receptors, activates the Janus kinase (JAK)-signal transducer and activator of transcription (STAT) pathway in a manner that directly represses COX-2 expression. Our results indicate that IFNT diminishes PLA2G4A expression, which would, in turn, reduce arachidonic acid release potentially resulting in diminished COX-2 expression and PG production. As indicated above, COX-2 and PLA2G4A gene expression may be co-regulated since the promoter region of each share common elements, including IFN response elements [12,32].

Results from the present study with BEND cells differ from results of our previous study with bovine luminal endometrial epithelial cells [19]. Similar to studies by others [14] BEND cells produced 10–20 fold more PGE_2 _than PGF_2α_, whereas luminal endometrial epithelial cells produce greater quantities of the latter. PDBu stimulation of BEND cell PG production did not affect PLA2G6 expression or activity and the PLA2G6 inhibitor, bromoenol lactone, did not diminish BEND cell PG production. Conversely, stimulation of PGF_2α _production with oxytocin in luminal endometrial epithelial cells increased PLA2G6 expression and activity and this response was attenuated by bromoenol lactone. In BEND cells, the PLA2G4A inhibitor, PYR-1, diminished PG production in unstimulated cells and abolished PDBu stimulation of PG production whereas it had little effect on luminal endometrial epithelial cell PG production (Ochs, Roberts, Elgayyer, Godkin and Tithof, unpublished observation). Together, these results indicate that PG production by the two cell types may be regulated by different PLA_2 _isotypes. Another possible explanation for the discordant results may be that stimulation with a phorbol ester may result in activation of signal transduction pathways different from those activated by oxytocin.

## Conclusion

PGE2 and PGF2-alpha production by BEND cells is mediated by the activity and expression of PLA2G4A. Interferon-tau treatment diminished expression of PLA2G4A and PG production. BEND cells were shown to express PLA2G6 but, unlike primary or early passage luminal bovine endometrial cells, stimulation of PLA2G6 activity was not associated with increased PG production. Identification of PLA_2 _enzymes as key regulators of PG production may lead to novel methods of regulating prostanoid production.

## Competing interests

The authors declare that they have no competing interests.

## Authors' contributions

JDG designed the study, overall, and drafted the manuscript. MPR performed the PG assays. WG and ME performed the PLA_2 _and Western blot assays. PKT supervised the assays and helped draft the manuscript. All authors read and approved the manuscript.
